# Long-Standing Temporomandibular Joint Dislocation: A Comprehensive Review and Proposal of a Treatment Algorithm

**DOI:** 10.3390/medicina61091505

**Published:** 2025-08-22

**Authors:** Kazuya Yoshida

**Affiliations:** Department of Oral and Maxillofacial Surgery, National Hospital Organization, Kyoto Medical Center, 1-1 Mukaihata-cho, Fukakusa, Fushimi-ku, Kyoto 612-8555, Japan; yoshida.kazuya.ut@mail.hosp.go.jp; Tel.: +81-75-641-9161

**Keywords:** temporomandibular joint dislocation, long-standing, protracted, chronic, reduction, treatment, surgery, algorithm

## Abstract

*Background and Objectives*: Long-standing or protracted temporomandibular joint dislocation refers to a condition that persists for more than 1 month without reduction. To elucidate the clinical characteristics and treatment results of this condition, this comprehensive review analyzed the available data. *Materials and Methods*: Studies were assessed using electronic medical databases and manual searches from their inception to 31 December 2024. *Results*: Overall, 229 cases (139 women and 81 men; mean age, 52.3 years) from 113 reports were assessed. The proportion of patients with bilateral and unilateral dislocations was 74.7% and 8.7%, respectively. The mean duration since dislocation was 11.9 months. Closed and open reductions were possible in 49 (21.4%) and 175 patients (76.4%), respectively. The mean dislocation duration was significantly (*p* = 0.001) shorter in patients who underwent closed reduction (4.9 months) than in those who underwent open reduction (14.8 months). *Conclusions*: Clinically, it is crucial to diagnose and treat this condition early to prevent it from becoming chronic. However, for cases of long-standing dislocations due to other more serious diseases, conservative treatments such as manual reduction and continuous elastic traction should be attempted first. If reduction fails, surgical treatment should be performed as an alternative.

## 1. Introduction

Dislocation of the temporomandibular joint (TMJ) refers to displacement of the mandibular condylar head from its normal position in the glenoid fossa. Dislocation of the TMJ is common, with an estimated annual incidence ranging from 2.5 to 25 cases per 100,000 individuals annually [1,2]. TMJ dislocations can be categorized as anterior, posterior, medial, lateral, or superior [3]. Anterior dislocations are the most frequently encountered, whereas posterior, medial, and lateral dislocations are comparatively rare. A systematic review reported 79 acute, 35 chronic, and 311 recurrent cases of TMJ dislocation [3].

The first recorded manual reduction of TMJ dislocations was described in the Edwin Smith papyrus, dating back to the 17th century BC [4]. The Edwin Smith papyrus is a record of a surgical procedure translated into English in 1930 [4]. This reduction method is not significantly different from the current method. Hippocrates also reported the method for anterior dislocations in the *Hippocrates Corpus* in the 5th century BC, which remains widely used today as the Hippocratic method. The Hippocratic method is the most widely recognized technique for manually reducing anterior TMJ dislocations. In this approach, the physician positions the thumbs laterally adjacent to the molars and places the remaining fingers under the mandible, applying downward pressure, followed by backward pressure to reposition the joint [1].

The European Society of Temporomandibular Joint Surgeons recently published consensus- and evidence-based guidelines for managing anterior condylar dislocations [2]. These guidelines recommend that the initial treatment should involve manual reduction using the Hippocratic method. If this approach fails, subsequent attempts may be made with the aid of medications, such as muscle relaxants and/or analgesics, and if required, under sedation or general anesthesia [2]. Securing methods should be considered for patients with recurrent, long-standing, and/or habitual dislocations. Non-surgical methods should be exhausted before attempting minimally invasive or open surgical interventions [2]. Botulinum toxin has been proposed as a potential therapeutic option for managing recurrent TMJ dislocations [5,6]. Indications for open surgical treatment [7,8,9] should be established only after the failure of conservative treatment and/or minimally invasive therapy for anterior TMJ dislocation [2].

Terms such as chronic, protracted, long-standing, prolonged, and permanent have been used to describe long-term, untreated TMJ dislocations. In this review, the author employs the term “long-standing”. No consensus exists regarding the duration after which an untreated dislocation should be considered long-standing. However, as most reports specify 1 or more months, this review adopts this definition.

Although a consensus has been reached regarding the treatment of anterior TMJ dislocation, the incidence of long-standing dislocation is significantly lower than that of acute dislocation, and no consistent treatment method has been established. Therefore, this review comprehensively analyzes all available reports of long-standing TMJ dislocation regarding its causes, symptoms, treatment, and clinical course and proposes a treatment algorithm for long-standing TMJ dislocation.

## 2. Materials and Methods

### 2.1. Literature Review

A comprehensive literature search was performed using major electronic medical databases and specific keywords relevant to the topic ([“long-standing” OR “chronic” OR “protracted” OR “prolonged” OR “permanent” OR “irreducible” OR “unreduced” OR “persistent”] AND [“temporomandibular joint” OR “mandible” OR “condyle” OR “mandibular condyle”] AND [“dislocation” OR “luxation”] AND [“reduction” OR “treatment” OR “management” OR “approach”]). Additionally, a manual search was conducted to identify articles cited from related sources. All reports identified through electronic databases or manual searches without language restrictions and published up to 31 December 2024 were screened as previously described [10,11]. The inclusion criteria were reports of cases in which TMJ dislocations were not reduced for more than 1 month and that described key patient information including sex, age, etiology, clinical presentation, and treatment. Before 1900, radiography was rarely used to diagnose TMJ dislocations. Therefore, reports published before 1900 were excluded from this review. Duplicate reports of the same case, studies lacking essential diagnostic imaging findings, records with incomplete data, and studies that were irrelevant to the objectives of the study were excluded. All eligible reports were independently assessed and reviewed by the author.

### 2.2. Analysis

Fundamental clinical data were extracted and evaluated from the selected studies. These included age, sex, affected side, etiology, chief complaint, diagnostic imaging findings, duration of dislocation, maximal mouth opening, presence of an open bite, treatment methods, complications, follow-up periods, and sequelae.

### 2.3. Statistics

Binomial logistic regression analysis was performed to evaluate whether age, sex, and dislocation duration could serve as potential predictors for selecting the appropriate treatment approach (closed versus open reduction). Two-tailed unpaired *t*-tests and Mann–Whitney U tests were used to evaluate the differences between groups. The ratio of closed and open reduction was analyzed in six groups: 1 month or more but less than 2 months after dislocation, 2 months or more but less than 3 months, 3 months or more but less than 4 months, 4 months or more but less than 5 months, 5 months or more but less than 6 months, and 6 months or more after dislocation. The difference in the duration after dislocation between cases with closed reduction and those with open reduction was analyzed using the Mann–Whitney U test. The etiology was classified as traumatic (e.g., trauma, fall, or motor vehicle accident) or atraumatic (e.g., yawning or dental treatment), and differences were analyzed using an unpaired *t*-test. All statistical analyses were performed using SPSS for Windows (version 24.0; SPSS Japan, Inc., Tokyo, Japan). Statistical significance was set at *p* < 0.05.

## 3. Results

**Figure 1:**
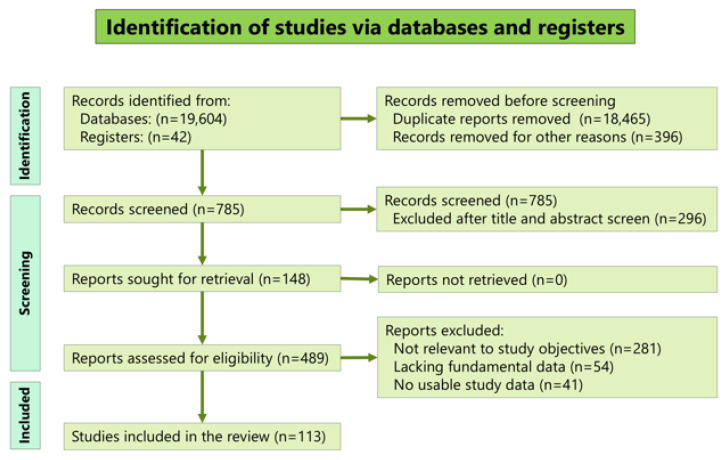
Diagram of the literature search and screening strategy.

The number of studies retrieved from electronic databases and registers, assessed for eligibility and included in the comprehensive review, is presented in a flow diagram (Figure 1). Overall, 19,604 articles were retrieved from the following databases: PubMed (313), Google Scholar (19,100), Japan Medical Abstracts Society (41), and J-Stage (150). Forty-two additional records were retrieved through manual searches of relevant papers and books. The search yielded 113 reports [12,13,14,15,16,17,18,19,20,21,22,23,24,25,26,27,28,29,30,31,32,33,34,35,36,37,38,39,40,41,42,43,44,45,46,47,48,49,50,51,52,53,54,55,56,57,58,59,60,61,62,63,64,65,66,67,68,69,70,71,72,73,74,75,76,77,78,79,80,81,82,83,84,85,86,87,88,89,90,91,92,93,94,95,96,97,98,99,100,101,102,103,104,105,106,107,108,109,110,111,112,113,114,115,116,117,118,119,120,121,122,123,124]. Table S1 shows the demographic data of all the patients. The number of evaluated reports categorized by the original language was as follows: English, 74; Japanese, 34; German, 4; and French, 1. The number of reports according to the number of cases was as follows: 1 case, 83; 2 cases, 13; 3 cases, 7; 4 cases, 2; 5 cases, 1; 6 cases, 1; 8 cases, 2; 10 cases, 1; 15 cases, 1; 19 cases, 1; and 20 cases, 1. All studies were case reports or case series.

### 3.1. Demographic Data and Diagnoses

A total of 113 articles comprising 229 patients were included in the analysis (mean age ± standard deviation: 52.3 ± 19.5 years; age range: 5–89 years) (Table S1). The demographic characteristics and diagnostic findings are summarized in Table 1. This study included 139 women (60.7%) and 81 men (35.4%). Among them, 171 (74.7%), 11 (4.8%), and 9 (3.9%) were bilateral, left-sided, and right-sided, respectively (Table 1). The mean duration of dislocation was 11.9 months (Table 1).

**Figure 2:**
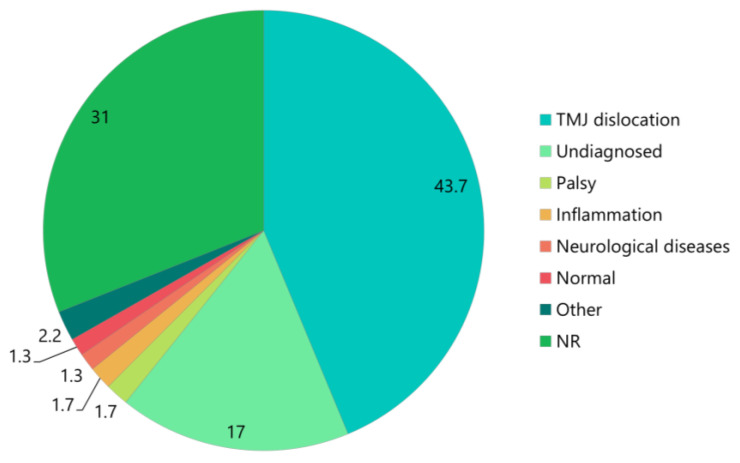
First clinical diagnosis of patients with long-standing TMJ dislocation. The numbers in the pie chart represent percentages. TMJ, temporomandibular joint; NR, not reported.

The initial clinical diagnosis was TMJ dislocation in 100 patients (43.7%). Additionally, four (1.7%), four (1.7%), and three (1.3%) patients were presented with palsy, inflammation, and neurological disease, respectively (Table 1, Figure 2).

Previous dislocations were reported once, frequently, and sometimes in 14 (6.1%), 6 (2.6%), and 3 (1.3%) patients, respectively (Table 1). Moreover, 37 patients (16.2%) reported no history of dislocation. In addition, the history of the dislocation itself was not recorded in 169 patients (73.8%).

The psychiatric diseases identified based on anamnesis included dementia or mental retardation, neurological diseases, cerebral infarction, cerebral hemorrhage, and brain injury in 15 (6.6%), 10 (4.4%), 10 (4.4%), 9 (3.9%), and 4 (1.7%) patients, respectively. No relevant neurological or psychiatric history was identified in 121 (52.8%) patients (Table 1).

Radiography was the primary diagnostic imaging modality (72.1%) (Table 1). Computed tomography (CT) and magnetic resonance imaging (MRI) were also performed (Table 1).

### 3.2. Symptoms and Etiology

The patients’ symptoms are summarized in Table 2. The most frequent chief complaints were preauricular pain (26.6%), inability to close the mouth (19.2%), masticatory disturbances (14%), and difficulty in speaking (10.5%) (Table 2).

The mean maximum mouth opening at the initial visit was restricted to 24.6 mm. The open bite (a gap between the anterior incisors when biting down) was 14.4 mm; however, it was reported in only 11.4% of the patients (Table 2).

**Figure 3:**
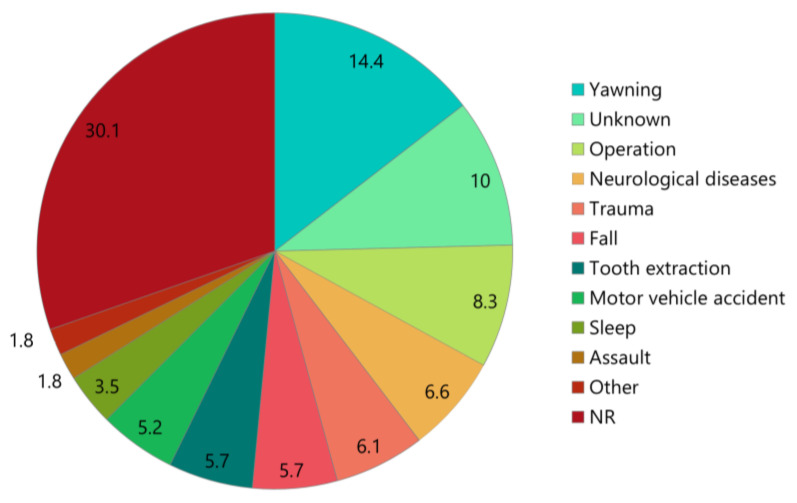
Etiologies of long-standing temporomandibular joint dislocation. NR, not reported.

The distribution of etiologies among all patients is shown in Figure 3. The most prevalent etiologies were yawning (14.4%, *n* = 33), surgical procedures (8.3%, *n* = 19), and neurological disorders (6.6%, *n* = 15). In 23 patients (10%), the etiologies were unknown, and 69 cases (30.1%) were not recorded (Table 2, Figure 3).

**Figure 4:**
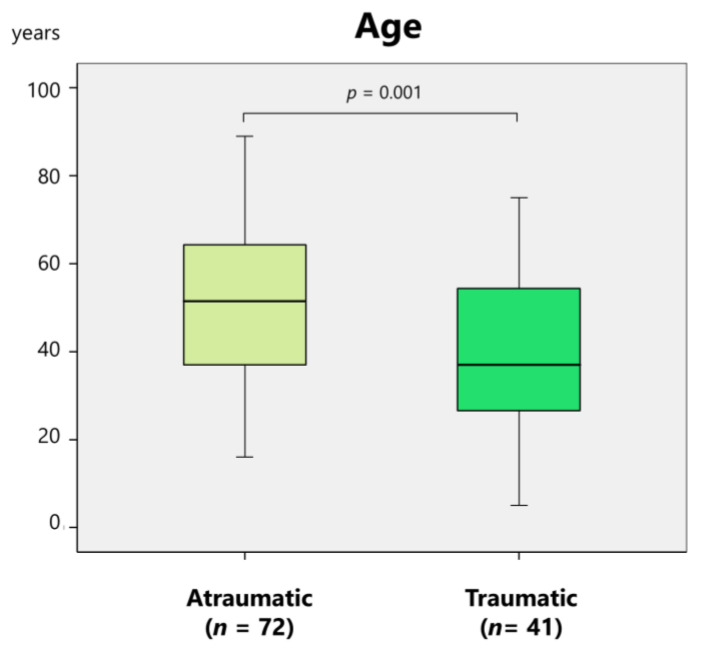
Comparison of the mean age of patients with atraumatic and traumatic etiologies.

Patients with traumatic etiology (*n* = 41) were significantly (*p* = 0.001) younger than those with atraumatic etiology (*n* = 72) (39.2 ± 17 years vs. 51.3 ± 39.2 years) (Figure 4).

### 3.3. Treatments and Sequelae

Table S2 presents the treatment outcomes, complications, follow-up periods, and sequelae for all patients. A summary of the treatment methods used in these patients is provided in Table 3. Local anesthesia, sedation, and general anesthesia were administered to 18 (7.9%), 51 (22.3%), and 189 (82.5%) patients, respectively (Table S2). Continuous traction with splints or wires was attempted in 29 patients (12.7%), 17 of whom (7.4%) achieved success without requiring an open procedure (Table 3). Additionally, closed manual reduction was performed in 183 patients (79.9%), of whom 28 (12.2%) showed successful outcomes (Table 3). Of these, 22, 5, and 1 were performed under general anesthesia, local anesthesia, and sedation, respectively.

**Figure 5:**
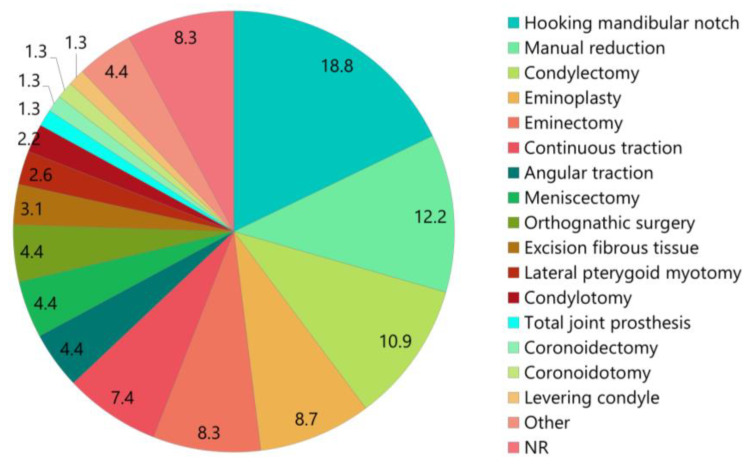
Treatments of patients with long-standing temporomandibular joint dislocation. NR, not reported.

Closed and open reductions were possible in 49 (21.4%) and 175 (76.4%) patients, respectively (Table 3 and Table S1). Surgical procedures included hook placement at the mandibular notch (18.8%), condylectomy (10.9%), eminoplasty (8.7%), eminectomy (8.3%), angular traction (4.4%), meniscectomy (4.4%), orthognathic surgery (4.4%), excision of fibrous tissue (3.1%), lateral pterygoid myotomy (2.6%), condylotomy (2.2%), total TMJ prosthesis (1.3%), coronoidectomy (1.3%), coronoidotomy (1.3%), and levering condyle (1.3%) (Table 3, Figure 5).

Regarding the approach used during the surgical procedure, a preauricular incision (46.7%) was the most frequent, followed by submandibular (10.5%) and zygomatic arch incisions (Table 3).

A total of 77 patients (66.4%) experienced no complications (Table 4). Post-reduction intermaxillary fixation was performed in 122 patients (53.3%) with an average duration of 17.5 days (Table 4). The average follow-up period was 11.8 months, ranging from 2 weeks to 96 months [99]; however, follow-up data were unavailable for 82 patients (35.8%). At follow-up, the maximal mouth opening was 36.2 mm (Table 4). The sequelae included redislocation (1.7%), deviation (1.3%), and condylar absorption (0.9%). No sequelae were observed in 55.9% of the patients, and data were not reported for 69 patients (30.1%; Table 4).

**Figure 6:**
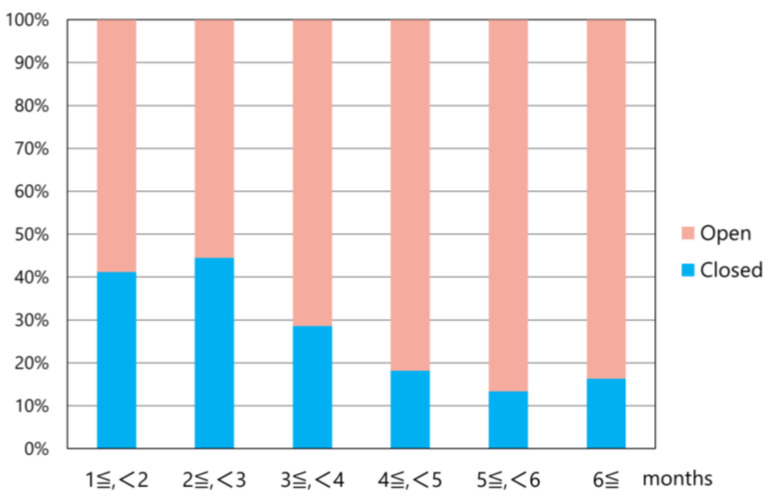
Ratio of closed and open reduction according to duration after dislocation.

Closed reduction was possible in more than 40% of cases within a 3-month duration; nonetheless, open reduction was required in more than 80% of cases after 4 months or more (Figure 6).

**Figure 7:**
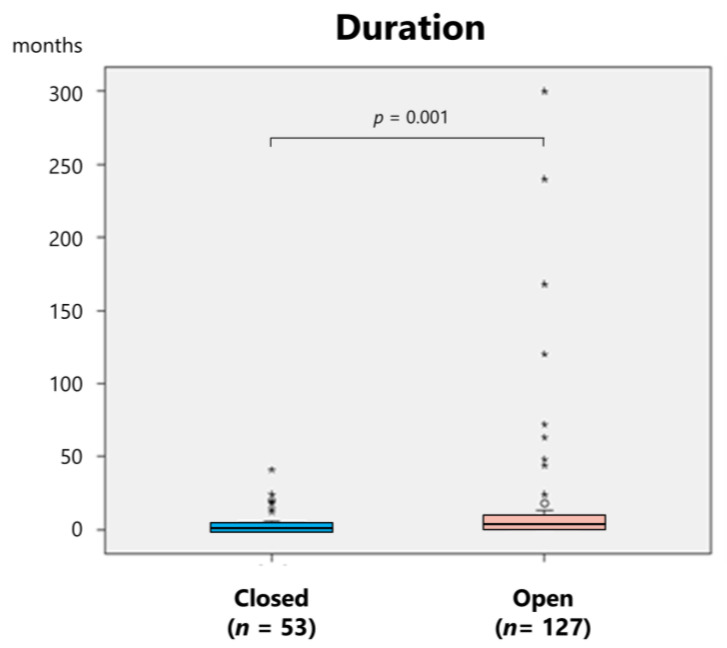
Duration of closed and open reduction in patients with long-standing temporomandibular joint dislocation.

The mean age of the patients who underwent closed and open reduction was 55.4 and 51.0 years, respectively, with no significant difference (*p* = 0.163, unpaired *t*-test). However, the time from dislocation to reduction was significantly shorter in patients treated with closed reduction than in those who required open reduction (4.9 ± 7.3 vs. 14.8 ± 40.4 months, respectively; *p* = 0.001, Mann–Whitney U test) (Figure 7).

Binomial logistic regression analysis was conducted to evaluate whether age, sex, or duration of dislocation could serve as predictors for determining the appropriate treatment approach (closed versus open procedures); however, no statistically significant factors were identified.

## 4. Discussion

This is the first extensive review of documented reports on long-standing TMJ dislocations. The clinical courses of 229 patients with long-standing TMJ dislocation that remained unreduced for more than one month were analyzed in this review. Although the early diagnosis and treatment of TMJ dislocation remain primary priorities, treatment is often delayed due to various factors. In cases involving dementia, mental retardation, or unconsciousness, the patients themselves cannot recognize the dislocation; therefore, medical and dental professionals must detect and diagnose it as early as possible. In cases of long-standing dislocation, treatment should proceed systematically, beginning with manual reduction before considering surgical options. However, there is no consensus regarding treatment policies or algorithms, which remains a significant challenge for future research.

### 4.1. Etiology of Long-Standing TMJ Dislocation

Reasons for TMJ dislocations remaining untreated for more than one month included patients being unaware of the dislocation due to dementia, impaired consciousness, or intellectual disability; edentulous patients not wearing dentures, making occlusal changes less apparent; postponement of TMJ dislocation treatment due to more serious life-threatening conditions; and patients not seeking medical attention, remaining undiagnosed, or being misdiagnosed (Table S1). This review revealed that the most prevalent etiologies of long-standing dislocations were yawning (14.4%), surgery (8.3%), neurological disease (6.6%), and unknown causes (10%) (Figure 2). In contrast, the common etiologies of superior condylar dislocation included motor vehicle accidents (50%), falls (20.7%), bicycle accidents (16.4%), and assault (3.4%) [11]. Compared with superior dislocations, the rate of traumatic etiology is notably lower. Long-standing TMJ dislocations can occur during routine movements such as yawning, and many cases have unknown or unspecified causes. These may result from minimal mouth opening beyond the normal range and often go undetected. Consequently, dislocations tend to recur during daily movements without trauma. If dislocation remains unrecognized due to dementia, mental retardation, psychiatric or neurological diseases, or unconsciousness, it can readily progress to a long-standing condition. In this study, patients with a traumatic etiology (*n* = 41) were significantly younger than those with an atraumatic etiology (*n* = 72) (39.2 years vs. 51.3 years) (Figure 4). The diagnosis and treatment of dislocations are often delayed in cases involving coexisting life-threatening injuries or conditions. Treatment decisions and prognoses may be influenced by factors such as the patient’s age, neurological involvement, and etiology.

Pseudoarticulation (nearthrosis) rarely develops in patients with long-standing dislocations when the dislocations persist for extended periods. Although lateral excursion is difficult to achieve, patients maintain a certain degree of function. To date, six cases of pseudoarticulation associated with long-standing TMJ dislocations have been reported [44,72,92,99,107,114].

Medical and dental professionals as well as caregivers should remain vigilant about dislocations in patients who cannot independently recognize abnormalities. Fortunately, recent advances in botulinum toxin therapy have eliminated the need for the surgical treatment of habitual TMJ dislocation when botulinum toxin is administered to the lateral pterygoid muscle following manual reduction [6,125]. If TMJ dislocation is promptly detected and treated, long-standing dislocation can be prevented, surgical treatment for TMJ dislocation will become largely unnecessary, and significant surgical intervention can be avoided [6].

### 4.2. Diagnosis of Long-Standing TMJ Dislocation

Generally, diagnosis is straightforward and based on clinical signs. The symptoms of bilateral TMJ dislocation include anterior mandibular protrusion, facial elongation, loss of both nasolabial folds, and preauricular depression with anterolateral prominence. Difficulty closing the mouth leads to drooling, impaired speech and pronunciation, and compromised chewing and swallowing. In unilateral dislocation, all symptoms manifest on one side, with mandibular deviation to the unaffected side and a crossbite. Radiography is the primary diagnostic imaging modality. MRI and CT may provide valuable diagnostic information when surgical procedures for the TMJ are necessary.

In most cases, early anterior TMJ dislocations can be reduced manually with relative ease [2]. However, if left untreated for more than one month, the likelihood of successful manual reduction decreases substantially, and the probability of requiring surgical procedures increases (Figure 5). Therefore, the early detection, diagnosis, and treatment of TMJ dislocations are crucial. However, many medical professionals are unfamiliar with TMJ dislocations. This review revealed that numerous cases progressed to chronicity due to missed diagnoses or misdiagnoses (Figure 2). When mouth closure or swallowing becomes difficult, and mouth breathing becomes necessary, the risk of aspiration pneumonia increases. Various neurological diseases cause involuntary contraction of the masticatory muscles, particularly the lateral pterygoid muscle [6,126,127,128]. Aspiration pneumonia is the most common cause of death in many neurological diseases, and untreated TMJ dislocation may contribute significantly to mortality. Oral surgeons and dentists with TMJ expertise should educate physicians, nurses, and other medical professionals about TMJ dislocations.

### 4.3. Treatment of Long-Standing TMJ Dislocation

Early diagnosis and treatment are essential for managing TMJ dislocation; however, when the dislocation becomes long-standing, treatment should progress gradually from non-invasive to invasive methods. Manual reduction (using analgesics, muscle relaxants, and local anesthesia as needed), manual reduction under sedation or general anesthesia, and continuous elastic traction using the lever technique should be attempted initially. Continuous traction using splints or wires was attempted in 12.7% of the cases; however, success was achieved without open procedures in only 7.4% of the cases (Table 3). Closed manual reduction was successful in 12.2% of patients (Table 3). Closed reduction was achievable in more than 40% of cases within 3 months of dislocation; however, open reduction was required in more than 80% of cases after 4 months or longer (Figure 6). Furthermore, the duration since dislocation in cases where closed reduction was achievable was significantly shorter than that in cases requiring open reduction (4.9 vs. 14.8 months) (Figure 6).

There are reports of closed reduction even in cases of prolonged dislocation. Caminiti and Weinberg [72] reported on the successful manual reduction of a unilateral TMJ dislocation under general anesthesia that persisted for 2 years. Ogawa et al. [97] successfully performed conservative reduction through lever action using a resin splint in a case of long-standing dislocation that persisted for 3 years and 5 months. Therefore, closed reduction appears feasible even in cases of extra-long-standing dislocations persisting beyond 6 months. However, when reduction using conservative methods is impossible, surgical procedures must be considered. Here, surgical procedures included hook placement at the mandibular notch (18.8%), condylectomy (10.9%), eminoplasty (8.7%), eminectomy (8.3%), angular traction (4.4%), meniscectomy (4.4%), orthognathic surgery (4.4%), excision of fibrous tissue (3.1%), lateral pterygoid myotomy (2.6%), condylotomy (2.2%), total TMJ prosthesis (1.3%), coronoidectomy (1.3%), coronoidotomy (1.3%), and condylar levering (1.3%) (Table 3, Figure 4). The Fink method involves the placement of a hook at the mandibular notch. A strong steel hook is inserted over the mandibular notch, and force is applied in the downward and backward directions to reduce the displaced condyle. McGraw first introduced this method in 1899 [129].

When manual reduction is not possible due to bony adhesions, alternatives such as condylectomy, condylotomy, or craniotomy should be considered. Gottlieb recommended condylectomy as a surgical procedure for long-standing TMJ dislocation because this technique is often necessary to prevent ankylosis [22]. Mazzoni first used this method to treat long-standing TMJ dislocations in 1877 [129].

Although eminectomy or eminoplasty has been applied primarily for habitual TMJ dislocation [130,131,132], both eminectomy [52,55,58,59,67,72,98,99,101,106,108,110,116,119] and eminoplasty [66,68,69,85,111,117] have also been utilized for long-standing dislocations.

Mandibulotomy enables the independent movement of each condyle, thereby eliminating resistance on the contralateral side [82,90,105]. Although no such cases have been reported, if reduction remains unsuccessful using this technique due to severe fibrous or bony adhesions around the TMJ, an approach to the TMJ may become necessary. The mandibular swing procedure utilizing midline mandibulotomy has been successfully used to access oral and oropharyngeal tumors. The advantage of midline mandibulotomy is the prevention of surgical exposure of the bilateral TMJs, facial scarring, and nerve injuries. Potential complications include lingual hematoma, damage to proximal root apices, malocclusion, and nonunion [90]. Reports are limited, and appropriate case selection requires careful consideration.

Orthognathic surgery is considered appropriate when closed reduction proves impossible, more than 6 months have elapsed since dislocation, and oral function remains maintained to a certain degree with the condyle in a dislocated position [20,36,44,67,81,103,114,124].

TMJ prostheses are gaining popularity and were applied in five cases across four reports included in this review [51,92,113,114]. According to a recent systematic review, TMJ prostheses are recommended only for patients presenting with severe ongoing pain, bony or fibrous ankylosis, or osteomyelitis following primary closed or open reduction of mandibular condyle fractures [133].

Although lateral pterygoid muscle myotomy has been used for recurrent or long-standing dislocations in several reports [12,73,107,116,134,135,136], botulinum toxin therapy may render myotomy largely unnecessary [11,126].

After successful reduction, intermaxillary fixation is required to preserve occlusion and avoid recurrence. Intermaxillary fixation was applied post-reduction in 122 patients (53.3%) for a mean duration of 17.5 days (Table 4). Redislocation has been reported by several researchers [36,58,74,87,108].

### 4.4. Limitations and Future Directions

**Figure 8:**
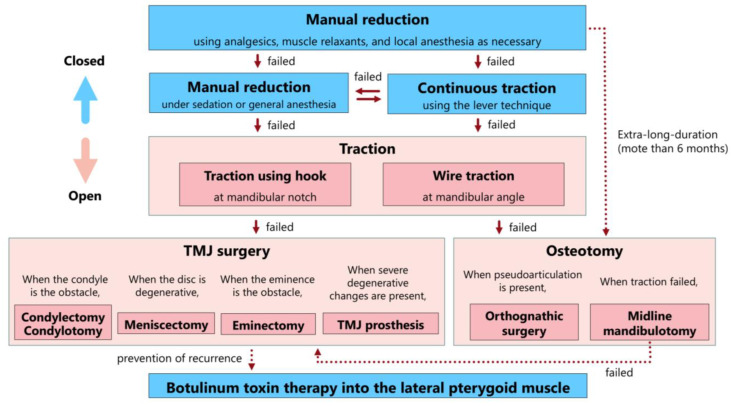
A proposed treatment algorithm for long-standing temporomandibular joint dislocation.

Follow-up data were not available for 35.8% of patients. The mean follow-up period was only 11.8 months. Future research requires sufficient longitudinal follow-up to determine postoperative sequelae. Further investigations, including a larger case series, are necessary to properly analyze the significant factors affecting outcomes. No significant factors were identified through binary logistic analysis, no general treatment policy existed, and treatment was implemented at the discretion of the attending physician. The author proposes an algorithm for managing and treating long-standing TMJ dislocations (Figure 8) based on the results of this review, previously suggested algorithms [84,90,99], and personal experience. Nonetheless, the rarity of this condition means that a consensus and robust evidence are lacking. Patient stratification and the corresponding selection of treatment methods are important when proposing an algorithm. However, previous studies have rarely described these aspects. To date, several clinicians have proposed various algorithms [84,90,99]. This review examined algorithms based on numerous studies including methods that have recently been applied and presented the most appropriate approach. However, this algorithm needs to be further developed and revised in the future to incorporate the opinions of experienced TMJ specialists, oral surgeons, and plastic surgeons based on the latest research results. Diagnostic delays can significantly influence the choice between closed and open reduction. Early diagnosis is crucial to facilitate less-invasive procedures and reduce the risk of long-term sequelae. As such, establishing and optimizing a standardized management approach for chronic dislocations is a pressing need in the medical field.

## 5. Conclusions

Early diagnosis and treatment of TMJ dislocation are essential to significantly reduce progression to chronic dislocation and avoid surgical intervention. Therefore, TMJ specialists should educate medical professionals about TMJ dislocations to increase awareness. For long-standing TMJ dislocations, the treatment approach should advance from conservative to more invasive methods in progressive stages.

## Figures and Tables

**Table 1:** Summary of the demographic data and diagnosis.

Sex, (*n* [%)]	Women, *n* = 139 (60.7%); men, *n* = 81 (35.4%); NR, *n* = 9 (3.9%)
Age (years), [mean ± SD, range]	52.3 ± 19.5, 5–89
Affected side, (*n* [%)]	Bilateral, *n* = 171 (74.7%); left, *n* = 11 (4.8%); right, *n* = 9 (3.9%); NR, *n* = 31 (13.5%)
Duration (months), (mean ± SD, *n* [%], range)	11.9 ± 34.4, *n* = 174 (76%), range 1–300
First clinical diagnosis, (*n* [%])	TMJ dislocation, *n* = 100 (43.7%); undiagnosed, *n* = 39 (17%); palsy, *n* = 4 (1.7%); inflammation, *n* = 4 (1.7%); neurological diseases, *n* = 3 (1.3%); normal, *n* = 3 (1.3%); other, *n* = 5 (2.2%); NR, *n* = 71 (31%)
Previous dislocation, (*n* [%])	Once, *n* = 14 (6.1%); frequently, *n* = 6 (2.6%); sometimes, *n* = 3 (1.3%); N, *n* = 37 (16.2%); NR, *n* = 169 (73.8%)
Anamnesis, (*n* [%])	Psychiatric diseases, *n* = 19 (8.3%); dementia or mental retardation, *n* = 15 (6.6%); neurological diseases, *n* = 10 (4.4%); cerebral infarction, *n* = 10 (4.4%); cerebral hemorrhage, *n* = 9 (3.9%); brain injury, *n* = 4 (1.7%); NR, *n* = 121 (52.8%)
Diagnostic image, (*n* [%])	Radiography; *n* = 165 (72.1%); CT, *n* = 57 (24.9%); MRI, *n* = 4 (1.7%); NR, *n* = 45 (19.7%)

SD, standard deviation; N, no; NR, not reported; CT, computed tomography; TMJ, temporomandibular joint; MRI, magnetic resonance imaging.

**Table 2:** Summary of patient symptoms and etiology.

Chief complaint, (*n* [%])	Preauricular pain, *n* = 61 (26.6%); inability to close mouth, *n* = 44 (19.2%); masticatory disturbance, *n* = 32 (14%); difficulty in speaking, *n* = 24 (10.5%); malocclusion, *n* = 15 (6.6%); facial deformity, *n* = 12 (5.2%); difficulty in swallowing, *n* = 12 (5.2%); other, *n* = 5 (2.2%); NR, *n* = 87 (38%)
Maximal mouth opening (mm), (mean ± SD, *n* [%])	24.6 ± 69.9, *n* = 62 (27.1%); NR, *n* = 167 (72.9%)
Open bite (mm), (mean ± SD, *n* [%])	14.4 ± 6.6, *n* = 26 (11.4%); NR, *n* = 203 (88.6%)
Edentulousness, (*n* [%])	Total edentulous, *n* = 48 (21%); upper or lower, *n* = 8 (3%); N, *n* = 95 (41.5%); NR, *n* = 62 (27.1%)
Etiology, [*n* (%)]	Yawning, *n* = 33 (14.4%); unknown, *n* = 23 (10%); surgical procedures, *n* = 19 (8.3%); neurological diseases, *n* = 15 (6.6%); trauma, *n* = 14 (6.1%); fall, *n* = 13 (5.7%); tooth extraction, *n* = 13 (5.7%); motor vehicle accident, *n* = 12 (5.2%); sleep, *n* = 8 (3.5%); assault; *n* = 4 (1.7%); others, *n* = 4 (1.7%); NR, *n* = 69 (30.1%)

SD, standard deviation; N, no; NR, not reported.

**Table 3:** Summary of treatments for long-standing TMJ dislocation.

Treatment, (*n* [%])	**Closed reduction**, *n* = 49 (21.4%) Manual reduction, *n* = 28 (12.2%); with instruments, *n* = 3 (1.3%), continuous traction, *n* = 17 (7.4%), 3–40 days
	**Open reduction**; *n* = 175 (76.4%) Hook placement at the mandibular notch, *n* = 43 (18.8%), condylectomy, *n* = 25 (10.9%), eminoplasty, *n* = 20 (8.7%), eminectomy, *n* = 19 (8.3%), angular traction, *n* = 10 (4.4%), meniscectomy, *n* = 10 (4.4%), orthognathic surgery, *n* = 10 (4.4%), excision of fibrous tissue, *n* = 7 (3.1%), lateral pterygoid myotomy, *n* = 6 (2.6%), condylotomy, *n* = 5 (2.2%), total TMJ prosthesis, *n* = 3 (1.3%), coronoidectomy, *n* = 3 (1.3%), coronoidotomy, *n* = 3 (1.3%), levering condyle, *n* = 3 (1.3%), other, *n* = 10 (4.4%), NR, *n* = 19 (8.3%)
	**Incision** Preauricular, *n* = 107 (46.7%), submandibular, *n* = 24 (10.5%), zygomatic arch, *n* = 15 (6.6%), intraoral, *n* = 13 (5.7%), Al-Kayat Bramery, *n* = 4 (1.7%), Bockenheimer–Axhausen, *n* = 3 (1.3%), other, *n* = 5 (2.2%)

N, no; TMJ, temporomandibular joint; NR, not reported.

**Table 4:** Summary of follow-up and sequelae in patients with long-standing TMJ dislocation.

Treatment complication, (*n* [%])	Facial nerve paralysis, *n* = 8 (3.5%); redislocation, *n* = 3 (1.3%); others, *n* = 3 (1.3%); N, *n* = 77 (33.6%); NR, *n* = 17 (7.4%)
Fixation (days), (*n* [%], mean ± SD)	Y, *n* = 122 (53.3%), 17.5 ± 15; N, *n* = 20 (8.7%); NR, *n* = 76 (33.2%)
Follow-up (months), (mean ± SD, *n* [%], range)	11.8 ± 13, *n* = 147 (64.2%), 0.25–96; NR, *n* = 82 (35.8%)
Maximal mouth opening at follow-up (mm), (mean ± SD, *n* [%], range)	36.2 ± 6.8, *n* = 90 (39.3%), 22–51, 35; NR, *n* = 23 (10%)
Sequelae, (*n* [%])	Redislocation; *n* = 4 (1.7%), deviation; *n* = 3 (1.3%), condylar absorption; *n* = 2 (0.9%), other; *n* = 9 (3.9%); N, *n* = 128 (55.9%); NR, *n* = 69 (30.1%)

SD, standard deviation; Y, yes; N, no; TMJ, temporomandibular joint; NR, not reported.

## Data Availability

Not applicable.

## Supplementary Materials

The following information can be downloaded from https://www.mdpi.com/article/10.3390/medicina61091505/s1, Table S1. Demographic data and patient symptoms. Table S2. Treatment and follow-up data for all patients.

## Abbreviations

The following abbreviations are used in this manuscript:

BC	Before Christ
CT	Computed tomography
MRI	Magnetic resonance imaging
TMJ	Temporomandibular joint
