# Supplementary material for: Long-Standing Temporomandibular Joint Dislocation: A Comprehensive Review and Proposal of a Treatment Algorithm
Source: Medicina (Kaunas). 2025 Aug 22;61(9):1505. doi: 10.3390/medicina61091505 (PMC12471310; doi:10.3390/medicina61091505)
Supplement: Supplementary file 1 [file medicina-61-01505-s001.zip › medicina-3775587-Table S1.pdf]

Table S1. Demographic data and patient symptoms.

Author/Year of publication	Age	Sex	Side	Duration (months)	First diagnosis	Etiology	Previous dislocation	Anamnesis	Chief complaint	Diagnostic imaging	Edentulousness	Maximal mouth opening (mm)	Open bite (mm)
Kramer (1901) [12]	20	W	R	1.25	TMJ dislocation	blow to R face	NR	NR	difficulty in chewing and speaking	NR	N	NR	NR
Hildebrand (1902) [13]	27	W	Bi	NR	NR	extraction of 9 teeth	NR	NR	inability to close the mouth	radiography	N	30	NR
Willcutts (1927) [14]	34	M	Bi	3	bad teeth	operation under general anesthesia	NR	submental abscess	inability to close the mouth, malocclusion, masticatory disturbance	radiography	NR	NR	NR
Miyakoda (1931) [15]	NR	NR	Bi	3	NR	NR	NR	NR	NR	NR	NR	NR	NR
Schwartz (1940) [16]	NR	M	L	48	bad teeth	fall	NR	NR	jaw deviation, masticatory disturbance	radiography	N	NR	NR
Reiß (1940) [17]	28	M	Bi	9	jaw fracture	accident by concrete mixing machine	NR	NR	masticatory disturbance	radiography	N	one fingerbreadth	NR
Watanabe & Hagino (1942) [18]	62	W	Bi	2.5	TMJ dislocation	fall	once 20 years ago	NR	masticatory disturbance	NR	upper, lower	30	13
Müller (1946) [19]	39	W	Bi	3	TMJ dislocation	extraction of 16 teeth	NR	low intelligence	malocclusion	NR	N	NR	NR
Jones (1949) [20]	16	M	Bi	120	TMJ dislocation	tonsillotomy	NR	NR	facial appearance	radiography	N	NR	NR
Watanabe & Otake (1950) [21]	28	W	Bi	4.5	TMJ dislocation	intrapartum eclampsia	NR	eclampsia	inability to close the mouth, difficulty in eating	radiography	N	2 fingerbreadths	1.5 fingerbreadths
Gottlieb (1952) [22]	24	W	L	18	TMJ dislocation	unknown	NR	maxillary sinusitis	pain in the L TMJ	radiography	N	40	NR
	60	M	Bi	5	prognathism	extraction of the last teeth	NR	NR	inability to close the mouth	radiography	upper, lower	NR	NR
	62	M	Bi	5	no abnormalities	dental treatment	NR	NR	inability to close the mouth, difficulty in swallowing, excessive salivation, indistinct speech	radiography	N	25	NR
Matsumae (1952) [23]	41	M	Bi	20	TMJ dislocation	during sleep	NR	NR	NR	NR	N	NR	7
Campbell et al. (1952) [24]	51	W	Bi	9	no abnormalities	extraction of 27 teeth	NR	NR	severe pain in the condyles	radiography	upper, lower	50	NR
Curson (1959) [25]	26	W	Bi	1	TMJ dislocation	extraction of some teeth	NR	NR	inability to close the mouth	radiography	N	NR	NR
Whinery (1961) [26]	20	W	Bi	4	TMJ dislocation	traffic accident	NR	NR	inability to close the mouth	radiography	N	NR	NR
Berg (1962) [27]	31	W	Bi	4.3	TMJ dislocation	epileptic seizures	NR	epilepsy	inability to close the mouth, difficulty in chewing and speaking	NR	N	NR	NR
Litzow (1962) [28]	61	M	L	6	TMJ dislocation	indeterminate convulsive disorder	NR	N	inability to close the mouth	radiography	N	NR	NR
Glahn (1964) [29]	30	W	Bi	1.25	TMJ dislocation	extraction of all teeth	NR	NR	pain in both TMJs	radiography	N	NR	NR
	46	M	R	18	TMJ dislocation	fall	NR	NR	pain in the R TMJ	radiography	NR	NR	NR
Hogan & Nally (1964) [30]	64	M	Bi	2.5	TMJ dislocation	during sleep	NR	NR	difficulties to close the mouth, talking, and eating	radiography	N	NR	NR
Fordyce (1965) [31]	33	W	Bi	6	undiagnosed	extraction of teeth under general anesthesia	NR	NR	discomfort, masticatory disturbance	radiography	upper	NR	NR

Hayward (1965) [32]	34	W	Bi	4	undiagnosed	removal of the mandibular teeth	NR	NR	inability to close the mouth, pain in the preauricular and temporal regions	radiography	upper, lower	NR	NR
	63	M	Bi	3	TMJ dislocation	removal of the mandibular teeth	NR	chronic duodenal ulcer	pain in the TMJ	radiography	N	NR	NR
	22	W	Bi	4	undiagnosed	tonsillectomy under general anesthesia	NR	loss of vision in the right eye due to a traffic accident	inability to close the mouth, difficulty in eating and speech	radiography	N	10	NR
Topazian & Costich (1967) [33]	31	M	L	2.25	inflammation	collapse	NR	NR	swelling in the L preauricular region	radiography	N	5	NR
Kameyama et al. (1968) [34]	75	W	Bi	5	undiagnosed	endotracheal intubation	NR	Japanese encephalitis	inability to close the mouth, difficulty in eating and speech	radiography	upper, lower	43	16
Yoshida et al. (1970) [35]	23	M	Bi	2	TMJ dislocation	traffic accident	N	N	facial deformity, difficulty in eating, swallowing, and speech	radiography	N	42	12
	71	W	Bi	7	TMJ dislocation	yawning	once 4 months ago	heart failure, cerebral embolism	masticatory disturbance, dysarthria	radiography	lower	15	NR
	38	W	Bi	5	undiagnosed	epileptic seizures	N	epilepsy	difficulty in eating, swallowing, and speech	radiography	upper	27	NR
Rowe & Caldwell (1970) [36]	27	M	Bi	72	TMJ dislocation	strike on the chin during volleyball	NR	asthma, chronic schizophrenia	jaw deformity, masticatory disturbance	radiography	N	NR	NR
Ohto et al. (1970) [37]	23	W	Bi	4	undiagnosed	NR	NR	mental illness	NR	radiography	N	NR	NR
Okano et al. (1971) [38]	53	M	Bi	3	undiagnosed	stroke	NR	stroke	limited mandibular movement, masticatory disturbance	radiography	N	NR	NR
Sujaku et al. (1972) [39]	44	W	Bi	2	TMJ dislocation	endotracheal intubation	NR	uterine cancer, acute renal failure	inability to close the mouth, masticatory disturbance	radiography	N	40	13
Horii et al. (1973) [40]	34	W	L	72	TMJ dislocation	unknown	NR	N	discomfort in L TMJ, malocclusion	radiography	N	4 fingerbreadth	N
Rawls et al. (1973) [41]	31	M	Bi	18	TMJ dislocation	unknown	NR	chronic alcoholism	difficulty in speech	radiography	N	20	15
Gorman (1974) [42]	89	W	Bi	for weeks rather than months	TMJ dislocation	NR	NR	frailty, heart failure	difficulty in speech and swallowing	radiography	lower, upper	NR	wide
Hashimoto et al. (1976) [43]	65	W	Bi	6	undiagnosed	fall	NR	subarachnoid hemorrhage, hypertension	inability to close the mouth, malocclusion	radiography	N	30	NR
	27	W	Bi	1	TMJ dislocation	yawning	often for 4 months	schizophrenia	malocclusion	radiography	N	21	11
Adekeye et al. (1976) [44]	25	M	Bi	12	TMJ dislocation	yawning	NR	N	inability to close the mouth, masticatory disturbance	radiography	N	30	5
Sanders et al. (1979) [45]	73	W	L	5	TMJ dislocation	spontaneous	N	hypothyroidism, cataract	pain of TMJ, mandibular deviation, inability to close the mouth, masticatory disturbance	radiography	upper, lower	NR	NR
Littler (1980) [46]	54	W	Bi	1.75	Bell's palsy	during sleep	NR	NR	inability to open the mouth	radiography	upper, lower	NR	NR
Kawamura et al (1980) [47]	48	M	Bi	3	TMJ dislocation	yawning	twice in the last 3 years	stroke, hypertension	masticatory disturbance malocclusion	radiography	N	NR	5
Mizuno et al. (1980) [48]	79	W	Bi	2	TMJ dislocation	NR	once 10 years ago	N	difficulty closing the mouth	radiography	upper, lower	NR	NR
Prabhakara & Andhra (1980) [49]	35	W	Bi	5	TMJ dislocation	yawning	NR	NR	difficulty with deglutition and speech	radiography	N	NR	NR
Stakesby Lewis (1981) [50]	NR	NR	NR	longer than 2 months	NR	NR	NR	NR	NR	NR	NR	NR	NR
	NR	NR	NR		NR	NR	NR	NR	NR	NR	NR	NR	NR
	NR	NR	NR		NR	NR	NR	NR	NR	NR	NR	NR	NR
	NR	NR	NR		NR	NR	NR	NR	NR	NR	NR	NR	NR
	NR	NR	NR		NR	NR	NR	NR	NR	NR	NR	NR	NR

	NR	NR	NR	4.75	NR	NR	NR	NR	NR	NR	NR	NR	NR
	NR	NR	NR	3	NR	NR	NR	NR	NR	NR	NR	NR	NR
	NR	NR	NR	many weeks	NR	NR	NR	NR	NR	NR	NR	NR	NR
Blank et al. (1982) [51]	37	M	Bi	18	TMJ dislocation	motorcycle accident	NR	NR	preauricular pain	radiography	N	35	NR
Tipps & Landis (1982) [52]	50	W	Bi	13	TMJ dislocation	NR	once	Crohn's disease, Paget's disease, cerebrovascular accident, chronic obstructive pulmonary disease, seizure disorder, drug withdrawal, anxiety, depression	Bi preauricular pain, inability to close the mouth	radiography	upper, lower	NR	NR
Parekh & Bhatia (1983) [53]	52	W	Bi	7	TMJ dislocation	yawning	once 8 months ago	N	inability to close the mouth, masticatory disturbance	radiography	N	33	25
Takahashi et al. (1984) [54]	74	W	Bi	1.3	TMJ dislocation	during sleep	NR	hypertension	inability to close the mouth	radiography	NR	NR	NR
	71	M	Bi	4	NR	tooth extraction	NR	N	inability to close the mouth, TMJ pain	radiography	upper, lower	NR	NR
Kudo et al. (1985) [55]	73	M	L	6	TMJ dislocation	NR	NR	L facial bone fracture, cataract	malocclusion, masticatory disturbance	radiography	upper, lower	25	NR
El-Attar & Ord (1986) [56]	47	W	Bi	9	Bell's palsy	during sleep	NR	NR	masticatory disturbance	radiography	upper, lower	NR	NR
Wijmenga et al. (1986) [57]	29	W	Bi	2	inflammation	teeth extraction	NR	NR	severe pain in TMJs	radiography	N	NR	10
	46	W	Bi	7	TMJ dislocation	extraction of wisdom tooth	NR	NR	masticatory disturbance, severe pain	radiography	N	NR	NR
Hammersley (1986) [58]	62	W	Bi	44	normal	fall	NR	NR	inability to wear full dentures	radiography	upper, lower	NR	NR
	49	W	Bi	uncertain	undiagnosed	yawning	NR	chronic schizophrenia with depression	NR	radiography	upper, lower	NR	NR
	NR	W	Bi	10	TMJ dislocation	NR	NR	Bell's palsy	malocclusion, masticatory disturbance	radiography	upper, lower	NR	NR
Suzuki et al. (1987) [59]	62	W	Bi	1.2	undiagnosed	vomiting	N	kidney stones	mandibular protrusion, inability of mouth opening	radiography	upper, lower	NR	NR
	74	W	Bi	1.9	inflammation	during sleep	N	hypertension, pneumonia	inability of mouth opening, masticatory disturbance	radiography	lower	NR	NR
Kowaka et al. (1987) [60]	69	W	Bi	19	undiagnosed	endotracheal intubation	NR	stroke, hemiplegia	difficulty in speech and swallowing	radiography	upper, lower	NR	NR
Obara et al. (1988) [61]	17	W	Bi	12	cerebral palsy	unknown	unknown	cerebral palsy, mental retardation	inability of mouth closing, pain in L ear region and masticatory disturbance	radiography	N	NR	26
Chin et al. (1988) [62]	59	M	Bi	10	TMJ dislocation	fall	NR	subdural hematoma	inability of mouth closing	radiography	upper, lower	NR	NR
Tanimoto et al. (1991) [63]	65	M	Bi	5	undiagnosed	endotracheal intubation	NR	intestinal obstruction, mental retardation	NR	radiography	lower	NR	NR
Ishihara et al. (1992) [64]	57	W	Bi	1.5	unknown etiology	endotracheal intubation	N	colon cancer, chronic renal failure, hypertension	inability of mouth closing, difficulty in speech	radiography	N	NR	9.6
Ishimaru et al. (1992) [65]	51	W	Bi	63	undiagnosed	unknown	frequently in the last 11 months	schizophrenia, mental retardation	inability of mouth closing	radiography	N	NR	NR
Ogawa et al. (1992) [66]	85	W	Bi	4	undiagnosed	unknown	unknown	senile dementia, bronchopneumonia	inability of mouth closing, difficulty in swallowing	radiography	upper, lower	NR	NR
Smith & Johnson (1994) [67]	57	M	Bi	4	acute dystonic reaction	unknown	NR	chronic schizophrenia	difficulty in swallowing, drooling of saliva	radiography	upper, lower	NR	NR
Kawakami et al. (1995) [68]	64	W	Bi	5	TMJ dislocation	unknown	frequently in the last 12 months	hemiplegia, intracranial arteriovenous malformation	masticatory disturbance	radiography	upper, lower	NR	NR

Kato et al. (1996) [69]	75	W	Bi	3	undiagnosed	unknown	NR	cerebral infarction, left hemiplegia, senile dementia	pain in the upper left molar area, inability of mouth closing	radiography	upper, lower	NR	NR
	66	W	Bi	5	undiagnosed	unknown	once 5 months ago	cerebral hemorrhage, cerebral infarction, senile dementia	masticatory disturbance	radiography	upper, lower	NR	NR
Iwatsubo et al. (1996) [70]	86	W	Bi	3	undiagnosed	unknown	NR	intracerebral hemorrhage, right hemiparesis	dysarthria, inability to close the mouth	radiography	upper, lower	NR	NR
Kurita et al. (1996) [71]	75	W	Bi	2	TMJ dislocation	fall	NR	NR	pain in the preauricular region, difficulty in eating and talking	radiography	NR	NR	NR
Caminiti & Weinberg (1998) [72]	73	W	R	120	malocclusion	unknown	NR	NR	malocclusion, masticatory disturbance	radiography	upper, lower	NR	NR
	16	W	L	24	undiagnosed	fall	NR	NR	facial asymmetry	radiography	N	NR	NR
	45	W	Bi	4	TMJ dislocation	fall	N	NR	inability to close the mouth	radiography	N	NR	24
Hoard et al. (1998) [73]	36	M	Bi	2.5	TMJ dislocation	motor vehicle accident	NR	NR	inability to close the mouth	radiography, CT	N	NR	NR
Mizutani et al. (2000) [74]	48	W	Bi	24	TMJ dislocation	yawning	frequently	psychiatric disorders	NR	radiography	N	NR	NR
	70	W	Bi	9	TMJ dislocation	unknown	N	cerebral infarction			N		
	64	W	Bi	3	TMJ dislocation	endotracheal intubation	N	cholelithiasis			upper, lower		
	62	M	Bi	2	TMJ dislocation	fall	once	N			N		
	56	M	Bi	2	TMJ dislocation	yawning	once	psychiatric disorders			N		
	72	W	Bi	6	TMJ dislocation	endotracheal intubation	N	cerebral infarction			upper		
	55	W	Bi	3	TMJ dislocation	endotracheal intubation	N	breast cancer			N		
	60	W	Bi	2	TMJ dislocation	endotracheal intubation	N	pancreatitis			upper, lower		
Murakami et al. (2002) [75]	67	M	R	6	TMJ dislocation	yawning	frequently in the last 6 years	Parkinson's disease, multiple cerebral infarctions	masticatory disturbance	radiography	N	NR	NR
Takenaka et al. (2003) [76]	83	W	Bi	1	TMJ dislocation	NR	once 60 years ago	Hypertension	inability to close the mouth	radiography, CT	upper, lower	NR	NR
Aquilina et al. (2004) [77]	71	M	R	2	undiagnosed	unknown	NR	L frontoparietal infarct, ischemic heart disease, chronic atrial fibrillation	limited jaw movement, facial pain	radiography	upper	NR	NR
Ohno et al. (2005) [78]	26	M	Bi	2	undiagnosed	traffic accident	NR	depression	inability to close the mouth	radiography	N	NR	15
Kobayakawa et al. (2005) [79]	77	M	Bi	2	undiagnosed	unknown	sometimes in the last 50 years	cerebral hemorrhage	inability to close the mouth	radiography	upper, lower	NR	NR
Terakado et al. (2006) [80]	81	W	Bi	Uncertain	TMJ dislocation	NR	NR	cerebral infarction	NR	radiography	N	NR	NR
Debnath et al. (2006) [81]	36	M	Bi	3	TMJ dislocation	yawning	NR	NR	pain in the ear region, inability to close the mouth	radiography	N	NR	NR
Lee et al. (2006) [82]	74	W	Bi	5	TMJ dislocation	traffic accident	NR	NR	swallowing disturbance, excessive salivation, indistinct speech	radiography, CT	upper	NR	NR
Yao et al. (2007) [83]	61	M	Bi	4	undiagnosed	unknown	NR	alcoholic liver failure, cirrhosis	masticatory disturbance, malocclusion	radiography, MRI	N	20	11
Rattan & Rai (2007) [84]	5	W	Bi	1	TMJ dislocation	fall	N	NR	facial pain, inability to close the mouth	radiography, CT	N	NR	NR

	70	W	Bi	1.3	TMJ dislocation	NR	recurrent dislocation	NR	inability to close the mouth	NR	upper, lower	NR	NR
	22	M	Bi	3	TMJ dislocation	road accident	NR	NR	pain in the temporal region, difficulty in chewing and mouth closing	CT	N	NR	6
	40	M	Bi	1.4	TMJ dislocation	road accident	NR	NR	NR	CT	N	NR	NR
Nakashima et al. (2007) [85]	58	W	R	240	undiagnosed	assault	N	bilateral tinnitus, sensorineural hearing impairment	malocclusion, pain in the R TMJ, facial deformity	radiography	N	NR	NR
Kale et al. (2010) [86]	48	W	Bi	2	TMJ dislocation	NR	NR	NR	dysarthria, masticatory disturbance, inability to close the mouth	radiography	N	1.5 fingerbreadth	NR
Huang et al. (2011) [87]	75	M	Bi	2	TMJ dislocation	NR	NR	systematic disease	NR	NR	NR	NR	NR
	75	M	Bi	2	TMJ dislocation	NR	NR	systematic disease	NR	NR	NR	NR	NR
	72	W	Bi	1	TMJ dislocation	NR	NR	chronic obstructive pulmonary disease	inability of closing mouth	NR	upper, lower	NR	NR
	68	M	Bi	2	TMJ dislocation	NR	NR	chronic obstructive pulmonary disease	NR	NR	upper, lower	NR	NR
	33	M	Bi	3.5	pericoronitis, cellulitis	NR	NR	sleep disorder	bite change	CT, MRI	N	NR	NR
	54	M	L	5	TMJ dislocation	traffic accident	NR	NR	mandibular deviation	radiography	N	NR	NR
Shakya et al. (2010) [88]	46	W	Bi	4	TMJ dislocation	teeth extraction	NR	psychiatric illness	inability to close the mouth, difficulty in talking	radiography	upper, lower	NR	NR
Kim & Kim (2012) [89]	74	W	Bi	6	TMJ dislocation	NR	once 2 years ago	thyroid cancer	L TMJ pain, masticatory disturbance	radiography, CT	upper, lower	NR	NR
Rattan et al. (2013) [90]	55	W	Bi	3	TMJ dislocation	vomiting	N	NR	inability to close the mouth	radiography	N	NR	NR
	40	M	Bi	1	TMJ dislocation	fall	NR	NR	inability to close the mouth	radiography	N	NR	NR
Yoshida et al. (2013) [91]	65	W	Bi	4	aftereffects of cerebral infarction	unknown	NR	cerebral infarction, atrial fibrillation, R hemiplegia	TMJ dislocation	radiography	upper, lower	NR	NR
Baur et al. (2013) [92]	73	W	Bi	4	stroke	yawning	N	NR	inability to close the mouth	radiography, CT	upper, lower	NR	NR
Elmorsy (2014) [93]	54	W	Bi	168	TMJ dislocation	trauma	NR	NR	severe mandibular protrusion, malocclusion	radiography	N	45	NR
	37	W	Bi	6	TMJ dislocation	trauma	NR	long bones and vertebrae fractures	pain in the L TMJ	radiography	N	NR	NR
Hayashi et al. (2014) [94]	83	W	Bi	2	undiagnosed	endotracheal intubation	NR	gastric ulcer, colon cancer, hypertension	inability to close the mouth	radiography	upper, lower	NR	NR
Saikia (2014) [95]	38	M	Bi	2	undiagnosed	yawning	N	NR	pain in the preauricular region, difficulty in talking, eating, mouth closing	radiography	N	NR	NR
Pradhan et al. (2015) [96]	29	W	Bi	2	TMJ dislocation	NR	NR	NR	inability to close the mouth	radiography	N	NR	10
	62	W	Bi	3	TMJ dislocation	NR	NR	NR	inability to close the mouth	radiography	N	NR	NR
Ogawa et al. (2015) [97]	31	W	Bi	41	TMJ dislocation	endotracheal intubation	10 years ago	schizophrenia, ovarian tumor	masticatory disturbance	radiography, MRI	N	NR	NR
Arzul et al. (2015) [98]	79	W	Bi	12	TMJ dislocation	NR	NR	Meige syndrome	pain in muscles and TMJ, masticatory disturbance	CT	N	NR	NR
Marqués-Mateo et al. (2016) [99]	70	W	Bi	1.5	TMJ dislocation	NR	N	NR	NR	NR	upper, lower	NR	NR
	34	W	R	72	TMJ dislocation	NR	NR	NR	myofascial pain	radiography	N	NR	NR

	76	W	Bi	2	TMJ dislocation	during sleep	NR	NR	pain, occlusal denture mismatch	NR	upper, lower	NR	NR
	50	M	Bi	4	undiagnosed	endotracheal intubation	NR	brain tumor of the temporal lobe	Malocclusion	radiography	N	NR	NR
Jeyaraj & Chakranarayan (2016) [100]	64	W	R	3	TMJ dislocation	yawning	NR	NR	mandibular deviation to L, inability to close the mouth	radiography, CT	N	41	NR
Güngörmüş et al. (2016) [101]	56	W	Bi	4	facial paralysis	yawning	N	N	pain in the temporal area, inability to close the mouth	radiography, CT	N	NR	NR
Negishi & Shibasaki (2017) [102]	89	W	Bi	1	TMJ dislocation	endotracheal intubation	several times	subarachnoid hemorrhage, dilated cardiomyopathy, atrial fibrillation, Alzheimer's disease	inability to close the mouth	radiography, CT	N	NR	NR
Malik et al. (2017) [103]	50	W	Bi	5	TMJ dislocation	yawning	NR	NR	inability to close the mouth, difficulty in swallowing and speech	radiography	N	15	NR
	34	M	Bi	4	TMJ dislocation	shouting	NR	psychiatric illness	inability to close the mouth	radiography	N	NR	15
Shaban et al. (2017) [104]	19-81 (37.8 ± 18.1)	W: 12 M: 8	NR	1.5-22 (3.6)	NR	yawning: 13 (W: 9, M: 4), psychiatric condition: 2 (W), trauma: 2 (M), sleep: 1 (M), tooth extraction: 1 (W), alcoholism: 1 (M)	NR	NR	NR	NR	NR	NR	NR
Dhiman et al. (2018) [105]	40	M	Bi	2	TMJ dislocation	blow to the chin	N	NR	inability to close the mouth, difficulty in speaking and swallowing	CT	N	NR	NR
	45	W	L	2	NR	trauma from rubber ball	NR	NR	preauricular pain and mandibular deviation	NR	NR	NR	NR
	57	M	Bi	2	TMJ dislocation	trauma to the mandible	NR	NR	difficulty in chewing, inability to close the mouth	radiography	NR	NR	NR
Chin et al. (2018) [106]	24	M	Bi	2	TMJ dislocation	endotracheal intubation	NR	intracerebral hemorrhage	inability to close the mouth	radiography, CT	N	42	15
Gholami et al. (2018) [107]	50	W	Bi	4	TMJ dislocation	yawning	N	N	inability to close the mouth	radiography	upper, lower	NR	20
	70	W	Bi	8	TMJ dislocation	yawning	NR	N	inability to close the mouth	radiography	N	NR	12
	73	W	Bi	4	TMJ dislocation	during eating	NR	NR	inability to close the mouth	radiography	upper, lower	NR	NR
Segami et al. (2018) [108]	69	M	Bi	NR	NR	NR	NR	cerebral infarction, diabetes mellitus, dementia	inability of jaw movement	radiography, CT	NR	NR	NR
	76	M	Bi	NR	NR	NR	NR	Parkinson's disease, schizophrenia, aspiration pneumonia, dementia	NR	NR	NR	NR	NR
	66	W	Bi	NR	NR	NR	NR	brain tumor, cerebral infarction, diabetes mellitus, dementia	NR	NR	NR	NR	NR
	83	W	Bi	NR	NR	NR	NR	cerebral infarction, atrial fibrillation, dementia, epilepsy	NR	NR	NR	NR	NR
	76	M	Bi	NR	NR	NR	NR	schizophrenia, Alzheimer's dementia,	NR	NR	NR	NR	NR

								cerebral infarction					
Balaji & Balaji (2018) [109]	32-58 (39.8 ± 13.6)	W: 12 M: 7	Bi	19.3 ± 12.6	NR	NR	NR	NR	NR	radiography, CT	NR	12-25 (17.8 ± 2.1)	NR
Güven (2019) [110]	85	M	Bi	18	TMJ dislocation	unknown	NR	dementia, grand mal epilepsy	masticatory disturbance	radiography	upper, lower	26	NR
	80	W	Bi	14	TMJ dislocation	unknown	NR	schizophrenia, depression, cardiovascular problems	masticatory disturbance	radiography	upper, lower	NR	NR
Cuevas Queipo de Llano et al. (2020) [111]	52	M	L	3	TMJ dislocation	unknown	NR	myotonic dystrophy	inability to close the mouth	radiography, MRI	upper, lower	NR	NR
Karakida et al. (2020) [112]	53	W	Bi	12	undiagnosed	unknown	NR	schizophrenia	inability to close the mouth	CT, radiography	N	NR	23
Sarlabous & Psutka (2020)	51	M	Bi	12	undiagnosed	seizure episode	NR	Seizure	difficulty with mouth opening and chewing	CT, radiography	upper, lower	25	NR
	61	W	Bi	7	TMJ dislocation	unknown	N	schizophrenia	severe pain and difficulty with function	CT, radiography	N	NR	NR
Bavia et al. (2020) [114]	38	M	R	300	undiagnosed	hit with a soccer ball	N	N	severe pain in the face	radiography, CT	N	54	NR
Uetsuki et al. (2022) [115]	69	M	Bi	4	TMJ dislocation	endotracheal intubation	NR	craniotomy for traumatic acute subdural hematoma, traumatic subarachnoid hemorrhage, traumatic localized brain contusion	inability to close the mouth	radiography, CT	N	NR	NR
Nikunj et al. (2022) [116]	21	M	Bi	6	TMJ dislocation	involuntary oral movement	once	schizophrenia	inability to close the mouth, preauricular pain	radiography, CT	N	NR	NR
Anehosur et al. (2023) [117]	35	W	Bi	1	NR	traffic accident	N	NR	NR	radiography, CT	N	20	NR
	46	M	Bi	1.5		NR	NR				upper, lower	22	
	18	W	Bi	1.75		traffic accident	NR				N	21	
	25	W	Uni	6		post labor	NR				N	25	
	43	W	Bi	1.5		assault	NR				N	22	
	34	M	Uni	3		traffic accident	NR				N	20	
	44	W	Bi	1		NR	N				N	20	
	56	W	Bi	1		NR	N				upper, lower	20	
	38	M	Bi	6		cerebrovascular accident	NR				N	24	
	24	M	Bi	1.5		NR	N				N	21	
	39	W	Bi	4.5		cerebrovascular accident	NR				N	21	
	35	M	Uni	1.25		assault	NR				N	22	
	32	W	Bi	1		assault	NR				N	23	
	35	W	Bi	2.5		cerebrovascular accident	NR				N	24	
	46	W	Bi	1.5		NR	N				upper, lower	21	
Ekram et al. (2022) [118]	18	M	Bi	1	TMJ dislocation	trauma	N	NR	inability of mouth closing	radiography, CT	N	NR	20
	27	M	Bi	1.5	TMJ dislocation	trauma	NR	NR	NR	radiography, CT	N	NR	NR
Navaneetham et al. (2023) [119]	47	M	Bi	9	TMJ dislocation	trauma	NR	traumatic brain injury with extradural hematoma	inability to close the mouth	radiography, CT	N	NR	28
Gupta et al. (2023) [120]	47	W	Bi	3	TMJ dislocation	endotracheal intubation	NR	diabetes mellitus, renal failure	difficulty in mouth closing, speech, and eating	radiography, CT	N	NR	NR
Ogundipe et al. (2023) [121]	60	W	Bi	2	TMJ dislocation	yawning	NR	epilepsy	NR	radiography	N	NR	NR

Zou et al. (2024) [122]	77	W	Bi	2	undiagnosed	NR	N	craniocerebral injury	NR	radiography, CT	N	N	N
	61	W	Bi	1	undiagnosed		N	craniocerebral injury					
	54	M	Bi	1	undiagnosed		N	cerebral hemorrhage					
	51	W	Bi	2	undiagnosed		once	cerebral hemorrhage					
	88	W	Bi	3	undiagnosed		N	cerebral infarction					
	74	W	Bi	6	TMJ dislocation		N	NR					
	78	W	Bi	1	undiagnosed		N	Alzheimer's disease					
	62	W	Bi	2	undiagnosed		N	craniocerebral injury					
	58	W	Bi	1	TMJ dislocation		Y	NR					
	69	W	Bi	2	undiagnosed		N	cerebral hemorrhage					
Tanaka et al. (2024) [123]	79	W	Bi	1	undiagnosed	unknown	NR	L cerebral infarction, hypertension	inability to close the mouth, dysphagia, dysarthria	radiography, CT	NR	NR	NR
Yanagisawa et al (2024) [124]	62	M	Bi	5	TMJ dislocation	yawning	NR	Hypertension	inability to close the mouth	radiography, CT	N	40	9

M: men; W: women; Bi: bilateral; Uni: unilateral; L: left; R: right; Y: yes; N: no; NR: not reported; TMJ: temporomandibular joint; CT: computed tomography; MRI: magnetic resonance imaging.
