# Supplementary material for: Long-Standing Temporomandibular Joint Dislocation: A Comprehensive Review and Proposal of a Treatment Algorithm
Source: Medicina (Kaunas). 2025 Aug 22;61(9):1505. doi: 10.3390/medicina61091505 (PMC12471310; doi:10.3390/medicina61091505)
Supplement: Supplementary file 1 [file medicina-61-01505-s001.zip › medicina-3775587-Table S2.pdf]

Table S2. Treatment and follow-up data for all patients.

	Attempt to closed reduction	Sedation	Continuous traction	Closed vs Open	Treatment	Approach	Anesthesia	Complication	Fixation	Length of fixation (days)	Follow-up (months)	Maximal mouth opening at follow-up (mm)	Sequelae
Kramer (1901) [12]	Y	NR	N	open	cutting of the lateral pterygoid muscle and lateral ligament	along the lower edge of the zygomatic arch	local?	N	NR	NR	NR	normal	N
Hildebrand (1902) [13]	Y	Y	N	open	reduction using the elevator between the skull base and the condyle, combined with manipulation	along the zygomatic arch	general	N	NR	NR	NR	NR	NR
Willcutts (1927) [14]	Y	NR	N	open	reduction after exposure of both joints	angular	local	N	NR	NR	NR	NR	NR
Miyakoda (1931) [15]	Y	NR	N	open	reduction by hooking the sigmoid notch with a hook	along the lower edge of the zygomatic arch	local	N	NR	NR	NR	NR	NR
Schwartz (1940) [16]	Y	NR	N	open	reduction after removal of fibrous tissue and exostosis from the condylar neck	preauricular	general	facial paralysis	Y	14	24	38	N
Reiß (1940) [17]	NR	NR	N	open	reduction after excision of adhesions around condyles	Bockenheimer-Axhausen	local	NR	Y	46	3.5	2.5 fingerbreadth	slight L deviation during mouth opening
Watanabe & Hagino (1942) [18]	Y	N	N	open	reduction with traction using the elevator	along the lower edge of the zygomatic arch	local	N	N	N	4	3 fingerbreadth	N
Müller (1946) [19]	Y	N	N	closed	manual reduction	N	N	N	Y	7	NR	NR	NR
Jones (1949) [20]	NR	N	N	open	Kostecka's operation	intraoral	general	N	Y	42	NR	NR	NR
Watanabe & Otake (1950) [21]	Y	N	Y	open	meniscectomy	around the external auditory canal, behind the ear	local	NR	Y	10	0.75	N	NR
Gottlieb (1952) [22]	NR	NR	NR	open	condylectomy	Bockenheimer-Axhausen	local	N	Y	112	12	40	slight deviation
	Y	N	N	open	condylectomy	preauricular	general	N	NR	N	8	NR	N
	Y	N	N	open	condylectomy	Bockenheimer-Axhausen	general	pronounced postoperative edema	NR	N	3	40	paresis of the forehead
Matsumae (1952) [23]	Y	N	Y	closed	reduction by continuous elastic traction for 20 days	N	N	N	N	N	NR	NR	NR
Campbell et al. (1952) [24]	Y	N	N	open	condylectomy	preauricular	general	slight drooping of L eyebrow	Y	NR	NR	NR	NR
Curson (1959) [25]	Y	Y	N	open	reduction after removal of fibrous adhesions	preauricular	general	N	Y	7	NR	NR	NR
Whinery (1961) [26]	Y	N	N	open	condylectomy	preauricular	general	N	Y	26	48	NR	N
Berg (1962) [27]	Y	Y	N	open	reduction by hooking the sigmoid notch with a hook	preauricular	general	relapse due to epileptic seizures	Y	32	3	NR	N
Litzow (1962) [28]	Y	N	N	open	condylectomy	preauricular?	general	small hematoma	N	N	2	NR	N
Glahn (1964) [29]	Y	N	N	open	open reduction	NR	NR	NR	NR	NR	NR	NR	NR
	NR	N	N	closed	a set of splints for 1 month	N	N	NR	NR	NR	NR	NR	NR
Hogan & Nally (1964) [30]	Y	N	N	closed	manipulation using arch wires and corks between the posterior teeth	N	N	N	Y	28	NR	NR	NR
Fordyce (1965) [31]	Y	N	N	closed	manual reduction using a Mason's gag in the molar region	N	general	NR	Y	21	24	NR	N
Hayward (1965) [32]	Y	N	N	open	distraction of the condyle using a steel wire at the angle, combined with intraoral manipulation	submandibular	general	little edema	NR	NR	NR	NR	NR
	Y	N	N	closed	manual reduction	N	general	N	Y	7	7	NR	N
	Y	N	N	closed	manual reduction	N	general	partial reduction	Y	14	30	NR	N

Topazian & Costich (1967) [33]	Y	N	N	open	reduction after levering the condyle with periosteal elevators combined with manipulation	preauricular	general	NR	Y	14	6	NR	L mandibular deviation on protrusion
Kameyama et al. (1968) [34]	Y	N	N	open	reduction by hooking the sigmoid notch with a hook	along the lower edge of the zygomatic arch	local	N	Y	14	5	35	N
Yoshida et al. (1970) [35]	Y	Y	N	open	reduction by hooking the sigmoid notch with a hook	submandibular	general	N	Y	7	2	32	N
	Y	N	N	open	reduction by hooking the sigmoid notch with a hook	submandibular	local	N	Y	11	2	26	N
	Y	Y	N	open	reduction by hooking the sigmoid notch with a hook	submandibular	general	N	NR	NR	4.5	32	N
Rowe & Caldwell (1970) [36]	Y	N	N	open	oblique bilateral osteotomy, coronoidotomy	intraoral	general	N	Y	21	3	NR	relapse to anterior apertognathic occlusion due to psychiatric crisis
Ohto et al. (1970) [37]	Y	N	N	open	reduction by hooking the sigmoid notch with a hook, condylotomy	along the lower edge of the zygomatic arch, preauricular	general	NR	Y	55	24	35	N
Okano et al. (1971) [38]	NR	N	Y	open	elastic traction (3 days) after reduction by hooking the sigmoid notch with a hook	along the lower edge of the zygomatic arch	general	N	NR	NR	NR	NR	NR
Sujaku et al. (1972) [39]	Y	Y	N	open	reduction by hooking the sigmoid notch with a hook	along the lower edge of the zygomatic arch	local	N	Y	10	6	35	N
Horii et al. (1973) [40]	Y	N	N	open	reduction after traction on the mandible using a steel wire passed through the mandibular angle	submandibular	general	N	Y	NR	6	2.5 fingerbreadth	N
Rawls et al. (1973) [41]	Y	Y	N	open	modified bilateral oblique osteotomies of the ramus after traction on the mandible using a steel wire passed through the mandibular angle	modified Risdon, intraoral	general	N	Y	28	6	35	N
Gorman (1974) [42]	Y	Y	N	open	Ward condylotomy	preauricular	general	NR	NR	N	8	NR	death from heart failure
Hashimoto et al. (1976) [43]	Y	N	N	open	reduction by hooking the sigmoid notch with a hook	along the lower edge of the zygomatic arch	general	N	Y	31	3	40	N
	Y	Y	N	open	reduction by hooking the sigmoid notch with a hook	along the lower edge of the zygomatic arch	general	N	Y	21	3	35	N
Adekeye et al. (1976) [44]	Y	N	N	open	inverted L-shaped ramus osteotomy	submandibular	general	N	Y	56	6	35	N
Sanders et al. (1979) [45]	Y	N	N	open	condylotomy	hockey-stick preauricular	general	N	Y	21	14	40	N
Littler (1980) [46]	Y	N	N	closed	manual reduction	N	local	N	NR	NR	1.5	32	N
Kawamura et al (1980) [47]	Y	N	Y	closed	traction using a resin plate with occlusal blocks (16 days)	N	N	N	Y	10	8	37	N
Mizuno et al. (1980) [48]	Y	Y	N	open	reduction by hooking the sigmoid notch with a hook	along the lower edge of the zygomatic arch	local	N	Y	7	4	NR	N
Prabhakara & Andhra (1980) [49]	Y	N	Y	closed	traction using maxillomandibular wires with occlusal blocks (5 days)	N	N	N	Y	21	12	NR	N
Stakesby Lewis (1981) [50]	Y	NR	NR	closed	manual reduction under general anesthesia	N	general	NR	N	N	NR	NR	NR
				closed	manual reduction under general anesthesia	N			N	N			
				open	reduction by insertion of a lever between the zygoma and the sigmoid notch	preauricular			N	N			
				open					N	N			
				open					N	N			

				open	reduction by direct force to the condyle using a Bristow's elevator	temporal			Y	2			
				open					Y	2			
				open					Y	2			
Blank et al. (1982) [51]	Y	Y	N	open	condylectomy, eminectomy, insertion of Proplast-coated Vitallium condylar prosthesis	preauricular, Risdon	general	NR	Y	21	12	35	N
Tipps & Landis (1982) [52]	Y	N	N	open	eminectomy, condylectomy, meniscectomy	inverted L-shaped	general	N	Y	5	6	40	N
Parekh & Bhatia (1983) [53]	Y	N	N	open	condylectomy	Kelikian	general	N	NR	NR	24	45	N
Takahashi et al. (1984) [54]	Y	N	N	closed	manual reduction under general anesthesia	N	general	N	NR	NR	NR	NR	NR
	Y	N	N	open	reduction by removal of the disc and connective tissue within the mandibular fossa	preauricular	general	N	Y	5	24	NR	N
Kudo et al. (1985) [55]	Y	Y	N	open	eminectomy	preauricular	general	N	Y	21	4.8	30	N
El-Attar & Ord (1986) [56]	Y	N	N	open	reduction by traction on the mandible using traction hooks in the holes of the angle	submandibular	general	N	Y	21	2	NR	NR
Wijmenga et al. (1986) [57]	Y	N	Y	closed	reduction under general anesthesia after insertion of a splint with a fulcrum in the molar region (3 days)	N	general	N	Y	14	4	NR	N
	Y	N	N	open	condylectomy	preauricular	general	NR	Y	56	12	NR	N
Hammersley (1986) [58]	NR	N	N	open	detachment of the lateral pterygoid insertions	preauricular with short temporal extensions	general	NR	Y	28	24	NR	recurrent dislocations of L TMJ
	Y	Y	N	open	L: condylectomy<br>R: eminectomy	preauricular	general	N	Y	NR	NR	NR	NR
	Y	N	N	open	traction using hooks at each angle	submandibular	general	N	Y	14	24	NR	N
Suzuki et al. (1987) [59]	Y	N	N	open	eminectomy, reduction by hooking the sigmoid notch with a hook	preauricular	general	N	Y	7	9	38	N
	Y	N	N	open	eminectomy	preauricular	general	N	Y	8	6	30	N
Kowaka et al. (1987) [60]	Y	N	Y	closed	intraoral resin plates with built-up heels and extra oral traction (40 days)	N	general	N	Y	40	39	NR	N
Obara et al. (1988) [61]	Y	N	N	closed	refused treatment after unsuccessful manual reduction	N	general	N	N	N	NR	NR	NR
Chin et al. (1988) [62]	Y	N	N	open	condylectomy, coronoidectomy, suprahyoid myotomy	Risdon	general	acute respiratory distress	Y	14	10	25	N
Tanimoto et al. (1991) [63]	Y	N	Y	open	intermaxillary elastic traction, reduction by hooking the sigmoid notch with a hook, condylectomy	preauricular	general	N	Y	12	13	NR	N
Ishihara et al. (1992) [64]	Y	N	Y	closed	elastic traction using bite blocks (7 days)	N	N	N	Y	30	7	29	slight TMJ pain when opening wide
Ishimaru et al. (1992) [65]	Y	Y	N	open	intermaxillary fixation after arthroscopic lysis and lavage	N	general	N	Y	17	11	35	N
Ogawa et al. (1992) [66]	Y	Y	N	open	meniscectomy, eminoplasty (Dautrey)	Al-Kayat Bramery	general	N	Y	7	5	40	N
Smith & Johnson (1994) [67]	Y	N	N	open	eminectomy, rotational sagittal split ramus osteotomy	intraoral	general	N	NR	NR	6	NR	N
Kawakami et al. (1995) [68]	Y	N	N	open	meniscectomy, eminoplasty (Dautrey)	Al-Kayat Bramery	general	N	NR	NR	31	NR	N
Kato et al. (1996) [69]	Y	N	N	open	manual reduction, eminoplasty (Leclerc)	Al-Kayat Bramery	general	N	N	N	22	NR	N
	Y	N	N	open	eminoplasty (Leclerc)	Al-Kayat Bramery	general	N	N	N	14	NR	N
Iwatsubo et al. (1996) [70]	Y	N	N	open	meniscectomy	preauricular	general	N	N	N	0.25	26	N
Kurita et al. (1996) [71]	Y	N	Y	open	partially reduction by elastic traction for 7 weeks after failure by reduction by hooking the sigmoid notch with a hook, then fully	preauricular	general	NR	NR	NR	9	NR	N

					reseated by 9 months								
Caminiti & Weinberg (1998) [72]	Y	N	N	open	reduction by excision of fibrous connective tissue and disengagement of the pseudoarticulation, recurred on the following morning, and refused further treatment	preauricular	general	N	N	N	NR	N	N
	NR	N	N	closed	manual reduction under general anesthesia	N	general	N	Y	14	2	NR	N
	Y	Y	N	open	eminectomy and traction with hooks and elevators	preauricular	general	N	Y	35	12	NR	N
Hoard et al. (1998) [73]	Y	N	N	open	reduction using spread scissors, mouth props, and sigmoid notch retractors combined with upward manual pressure after Bi coronoidectomies and stripping the masseter, medial pterygoid, and lateral pterygoid muscles	intraoral posterior vertical buccal	general	NR	Y	14	NR	NR	NR
Mizutani et al. (2000) [74]	Y	N	N	open	reduction by hooking the sigmoid notch with a hook	along lower edge of zygomatic arch	general	N	Y	14		NR	redislocation
	Y	N	Y	open	reduction by hooking the sigmoid notch with a hook		general	N	Y	14		NR	condylar absorption
	Y	N	N	open	reduction by hooking the sigmoid notch with a hook		general	N	Y	14		NR	N
	Y	N	N	closed	manual reduction under general anesthesia	N	general	N	Y	14	6-24 (mean: 13.2)	NR	N
	Y	N	N	closed	manual reduction under general anesthesia	N	general	N	Y	14		NR	N
	Y	N	N	open	reduction by hooking the sigmoid notch with a hook	along lower edge of zygomatic arch	general	N	Y	14		NR	N
	Y	N	N	open	reduction by hooking the sigmoid notch with a hook		general	N	Y	14		NR	N
	Y	N	Y	open	reduction by hooking the sigmoid notch with a hook		general	N	Y	14		NR	condylar absorption
Murakami et al. (2002) [75]	Y	N	N	open	removal of adhesions and curettage	approach to sigmoid notch	general	N	Y	14	12	NR	N
Takenaka et al. (2003) [76]	Y	Y	N	closed	manual reduction after arthroscopic lysis and lavage	N	general	NR	N	N	8	38	N
Aquilina et al. (2004) [77]	NR	N	N	closed	manual reduction, botulinum toxin therapy for the lateral pterygoid and temporalis muscles	N	general	NR	Y	14	1.5	NR	N
Ohno et al. (2005) [78]	Y	N	N	closed	manual reduction after intra-articular pumping of the upper joint space of the TMJ	N	local	N	Y	7	5	40	N
Kobayakawa et al. (2005) [79]	Y	N	N	open	manipulative reduction after eminectomy and removal of fibrous adhesive lesions	preauricular	general	N	Y	7	12	34	suspected cortical bone fracture of the L condyle
Terakado et al. (2006) [80]	Y	N	Y	closed	anterior traction with elastics using acrylic bite blocks (22 days)	N	N	N	Y	14	NR	25	NR
Debnath et al. (2006) [81]	Y	N	N	open	vertical-oblique osteotomies of the ramus	external	general	NR	NR	NR	18	NR	N
Lee et al. (2006) [82]	Y	Y	N	open	midline mandibulotomy after failure by reduction by hooking the sigmoid notch with a hook	over the mandibular notch, intraoral	general	N	Y	10	24	NR	N
Yao et al. (2007) [83]	Y	Y	Y	closed	reduction by lever action using intraoral blocks with built-up heels and elastic traction (17 days)	N	N	N	Y	14	12	38	N
Rattan & Rai (2007) [84]	Y	N	N	open	wire traction at mandibular angle, direct open reduction after exposure of both TMJs	submandibular, preauricular	general	N	Y	21	8	35	slight resorption of the R condyle
	Y	N	N	closed	manual reduction under local anesthesia	N	local	N	Y	NR	NR	NR	NR
	Y	N	Y	open	elastic traction after wire traction at the mandibular angle	submandibular	general	N	Y	10	3	NR	N
	Y	N	Y	open	elastic traction after wire traction at the mandibular angle	submandibular, preauricular	general	N	Y	NR	3	NR	3 mm anterior open bite

Nakashima et al. (2007) [85]	NR	N	N	open	osteotomy of zygomatic arch, eminoplasty	preauricular, horizontal	general	N	Y	14	24	30	NR
Kale et al. (2010) [86]	Y	N	Y	closed	manual reduction using a Fergusson's mouth gag, retraction by chin cap (11 days)	N	local	N	N	N	NR	NR	NR
Huang et al. (2011) [87]	Y	N	N	closed	declined treatment after unsuccessful manual reduction	N	N	N	N	N	N	N	N
	Y	N	N	closed	declined treatment after unsuccessful manual reduction	N	N	N	N	N	N	N	N
	Y	Y	N	closed	manual reduction under sedation	N	N	N	Y	14	6	NR	N
	NR	NR	N	open	manipulation assisted by wire traction at the mandibular angles	submandibular	general	NR	Y	relapse on the second day, abandoned the treatment			
	Y	N	N	open	reduction using a wire at an angle	intraoral, submandibular	general	N	Y	28	1	NR	NR
	Y	N	N	closed	manual reduction under general anesthesia	N	general	N	Y	NR	3	NR	NR
Shakya et al. (2010) [88]	Y	N	N	closed	manual reduction under local anesthesia	N	local	N	Y	7	3	NR	NR
Kim & Kim (2012) [89]	Y	Y	N	open	condylectomy	preauricular	general	N	N	N	2	NR	NR
Rattan et al. (2013) [90]	Y	N	N	open	midline mandibulotomy after unsuccessful traction with wires at the angle	labial vestibular	general	NR	Y	7	24	NR	N
	Y	N	Y	open	midline mandibulotomy after unsuccessful traction with wires at the angle	labial vestibular	general	NR	Y	10	12	NR	N
Yoshida et al. (2013) [91]	Y	Y	Y	closed	traction using intermaxillary fixation screws (11 days)	N	N	N	Y	23	12	NR	N
Baur et al. (2013) [92]	Y	NR	N	open	condylectomy, reconstruction with alloplastic total joint prosthesis	preauricular, submandibular	general	NR	Y	NR	7	35	N
Elmorsy (2014) [93]	NR	N	N	open	sagittal split osteotomy	buccal mucosa	general	N	Y	21	12	NR	N
	NR	N	N	open	condylotomy	preauricular	general	NR	NR	N	12	NR	N
Hayashi et al. (2014) [94]	Y	Y	N	closed	manual reduction under general anesthesia	N	general	N	Y	27	18	NR	N
Saikia (2014) [95]	Y	N	Y	closed	elastic traction with a bite plate (7 days)	N	N	N	Y	7	NR	NR	NR
Pradhan et al. (2015) [96]	Y	N	Y	closed	elastic traction with posterior acrylic bite blocks for 14 days	N	N	N	Y	14	NR	NR	NR
	Y	N	Y	closed	elastic traction with posterior acrylic bite blocks for 21 days	N	N	N	NR	NR	NR	NR	NR
Ogawa et al. (2015) [97]	Y	N	Y	closed	conservative reduction by lever action (29 days)	N	N	N	Y	56	24	42	N
Arzul et al. (2015) [98]	Y	Y	N	open	eminectomy, botulinum toxin therapy into the masseter and temporalis muscles	preauricular	general	N	Y	7	18	NR	N
Marqués-Mateo et al. (2016) [99]	Y	N	N	open	pulling down on wires from both mandibular angles	NR	general	N	NR	NR	60	NR	N
	NR	N	N	open	condylotomy	retromandibular via Risdon, preauricular	general	NR	NR	NR	96	NR	N
	Y	N	N	closed	manual reduction under general anesthesia	N	general	N	Y	21	36	NR	NR
	Y	N	N	open	meniscectomy, eminectomy, high condylotomy	preauricular	general	NR	Y	14	12	NR	N
Jeyaraj & Chakranarayan (2016) [100]	Y	N	N	open	excision of fibrous tissue, disengagement of the pseudoarticulation, downward traction on the R mandibular angle with wire	preauricular	general	N	N	N	14	NR	N
Güngörmüş et al. (2016) [101]	Y	Y	N	open	eminectomy and chin-cap support	preauricular	general	NR	Y	42	6	38	N
Negishi & Shibasaki (2017) [102]	Y	N	Y	open	intraoral condylectomy, L coronoidotomy after traction with leverage	buccal mucosa	general	NR	Y	27	1.6	NR	NR
Malik et al. (2017) [103]	Y	N	N	open	wire traction at the mandibular angle, inverted "L" osteotomy	submandibular	general	N	Y	28	12	NR	N
	Y	N	N	open	wire traction at the mandibular angle, vertical osteotomy	preauricular	general	N	NR	NR	8	NR	N

Shaban et al. (2017) [104]	Y	Y	Y	open	traction at the mandibular notch using a Warwick-James elevator (19 cases), for a case that required condylectomy	preauricular	general	NR	NR	NR	1	NR	Y
Dhiman et al. (2018) [105]	Y	Y	N	open	R traction of angle, osteotomy of angle, condylectomy, L open reduction	submandibular, preauricular	general	NR	NR	NR	NR	NR	Y
	Y	Y	N	open	traction at the mandibular notch using a Langenbeck retractor	retromandibular	general	NR	NR	NR	NR	NR	Y
	Y	N	N	open	hemimandibulotomy	intraoral	general	NR	NR	NR	NR	NR	Y
Chin et al. (2018) [106]	Y	Y	Y	open	L emniscotomy, release of thick fibrous tissues around TMJ, cut of TMJ capsules	preauricular	general	NR	NR	NR	1	NR	N
Gholami et al. (2018) [107]	Y	Y	N	open	disengagement of pseudoarticulation, excision of all fibrous tissues, meniscectomy, lateral pterygoid muscle myotomy	submandibular, preauricular	general	NR	NR	NR	NR	NR	NR
	Y	Y	N	open	excision of all fibrous tissues	retromandibular	general	NR	NR	NR	NR	NR	NR
	Y	N	N	open	excision of all fibrous tissues, meniscectomy	intraoral?	general	NR	NR	NR	NR	NR	NR
Segami et al. (2018) [108]	Y	Y	N	open	R tethering single set, L eminectomy	bicoronal incision with preauricular	general	NR	Y	14	12	NR	N
	Y	N	N	open	R tethering double set, L tethering single set	preauricular	general	NR	Y	10	36	NR	N
	Y	N	N	open	R tethering single set, L tethering double set	preauricular	general	NR	NR	NR	36	NR	N
	Y	N	N	open	R tethering single set, L eminectomy	preauricular	general	NR	Y	10	36	40	N
	NR	NR	NR	open	Bi tethering single set	preauricular	general	NR	NR	NR	12	NR	died after 12 months
Balaji & Balaji (2018) [109]	NR	NR	NR	open	hook-shaped miniplates and miniscrews fixed with or without bone grafts	preauricular	local	NR	NR	NR	22	NR	recurrence 5 months after Bi eminectomy, died after 22 months
Güven (2019) [110]	NR	NR	NR	open	eminectomy	preauricular	general	NR	NR	NR	14	NR	died after 14 months
	NR	NR	NR	open	declined surgical intervention and was fitted with complete dentures	preauricular	general	NR	NR	NR	33	NR	died after 33 months
Cuevas Queipo de Llano et al. (2020) [111]	NR	NR	NR	open	discectomy, condylectomy, arthroplasty	preauricular	general	NR	NR	NR	42	NR	NR
Karakida et al. (2020) [112]	Y	NR	NR	open	condylectomy	preauricular	general	N	NR	NR	8-37	27-37 (32.3 ± 3.2)	N
Sarlabous & Psutka (2020) [113]	Y	NR	NR	open	total joint replacement with alloplastic devices	preauricular	general	NR	N	N	2	NR	NR
	Y	Y	N	closed	condylectomy, coronoidotomy, total joint replacement with alloplastic devices	preauricular, retromandibualar	general	mandibular nerve neuroplaxia	NR	NR	3	NR	NR
Bavia et al. (2020) [114]	Y	N	N	open	first: R eminectomy, condylectomy, meniscectomy, coronoidotomy, placement of the intermediate condylar prosthesis second: R placement of R TMJ prosthesis, L: sagittal split osteotomy	preauricular	general	N	N	N	12	36	N
Uetsuki et al. (2022) [115]	Y	N	N	open	R condylectomy, coronoidectomy	submandibular	general	N	N	N	NR	NR	NR
Nikunj et al. (2022) [116]	NR	N	N	open	eminectomy, lateral pterygoid myotomy	preauricular, retromandibular	general	mild R temporal nerve weakness	NR	NR	36	35	N
Anehosur et al. (2023) [117]	NR	NR	NR	open	eminoplasty	preauricular	general	N	Y	7	12	35	NR
								N				36	
								facial nerve paralysis				35	
								N				35	
								N				35	
								N				35	
								infection				36	
								N				35	
								N				35	

								N				35	
								N				35	
								infection				35	
								N				35	
								N				36	
								facial nerve paralysis				35	
Ekram et al. (2022) [118]	Y	N	Y	closed	elastic contraction using posterior rubber bite block (21 days)	N	N	N	Y	21	NR	NR	NR
	Y	N	Y	closed	elastic contraction using posterior rubber bite block (22 days)	N	N	N	Y	NR	NR	NR	NR
Navaneetham et al. (2023) [119]	Y	N	N	open	eminectomy, condylectomy	preauricular	general	palsy of the zygomatic branch of the facial nerve	Y	17	2	35	NR
Gupta et al. (2023) [120]	N	N	Y	closed	anterior traction with elastics using Ehrich's arch wire with 10 mm bite blocks (15 days)	N	N	N	Y	10	NR	NR	NR
Ogundipe et al. (2023) [121]	Y	N	N	open	direct application of reduction forceps on the angle of the mandible	stab	general	N	Y	14	NR	NR	NR
Zou et al. (2024) [122]	Y	N	N	closed	manual reduction under general anesthesia	N	general	N	Y	21	6	NR	NR
				closed	manual reduction under general anesthesia, traction nail, and rubber bands	N	general						
				closed		N	general						
				closed		N	general						
				closed		N	general						
				closed		N	general						
				closed	manual reduction under local anesthesia	N	local						
				open	manual reduction under general anesthesia, traction nail, and rubber bands	anterioauricular crutch	general	facial nerve damage					
				closed		N	general	N					
				closed		N	general	N					
Tanaka et al. (2024) [123]	Y	Y	NR	closed	manual reduction under general anesthesia	N	general	NR	NR	NR	NR	NR	NR
Yanagisawa et al (2024) [124]	NR	NR	N	open	eminectomy, intraoral vertical ramus osteotomy	enauricular, intraoral	general	N	Y	5	36	35	N

L, left; R, right; Y, yes; N, no; NR, not reported; TMJ, temporomandibular joint.
